# Superluminal Tunneling and the Sauter–Schwinger Effect

**DOI:** 10.3390/e28060583

**Published:** 2026-05-23

**Authors:** Randall S. Dumont

**Affiliations:** Department of Chemistry and Chemical Biology, McMaster University, Hamilton, ON L8S 4M1, Canada; dumontr@mcmaster.ca

**Keywords:** Sauter–Schwinger effect, superluminal tunneling, Dirac equation, Klein zone, semiclassical WKB model, MacColl–Hartman effect

## Abstract

Previous 1+1-dimensional Dirac wavepacket calculations showed that the tunneling component of a relativistic electron wavepacket can generate an arrival-time distribution whose peak occurs earlier than the corresponding free-photon peak. However, adapting superluminal tunneling to signaling leads to subluminal signaling due to the low tunneling probability. In the present work we note that the barriers used in those calculations are supercritical with respect to the Sauter–Schwinger effect. Consequently, the single-electron evolution must be accompanied by spontaneous electron–positron production from the vacuum. We derive compact formulas for the electron and positron densities when one additional electron is present, showing that the evolved wavepacket contribution adds to the vacuum-produced electron density, while Pauli blocking reduces the positron density by the negative-energy component of the propagated electron. We then apply these formulas to a fourth-order super-Gaussian barrier which produces superluminal tunneling of an electron. The resulting densities are shown explicitly at several times, and are compared with a semiclassical resonance model for the pair number. The semiclassical description reproduces the numerical growth of the pair yield and clarifies the role of Klein-zone resonance energies and widths. Finally, we outline the extension from 1+1 to 1+3 dimensions by integrating over transverse momenta, using scaling properties of the 1+1-dimensional pair number.

## 1. Introduction

The possibility of apparent superluminal tunneling has been discussed since the early wavepacket work of MacColl and Hartman [1,2]. MacColl found that a transmitted wavepacket may emerge with very little delay relative to free motion, and Hartman later showed that, for sufficiently opaque barriers, the associated delay time can become almost independent of barrier width. In relativistic settings this behavior can become especially striking, because the transmitted component of a Dirac wavepacket may arrive with an effective velocity exceeding *c* when interpreted naively in terms of the initial wavepacket peak to tunneling-component peak distance divided by flight time.

In two earlier papers we studied this issue using Gaussian wavepackets propagated with the 1+1-dimensional Dirac Hamiltonian [3,4]. The first paper demonstrated tunneling arrival-time distributions that peak earlier than d/c, where *d* is the distance from the center of the initial Gaussian to the far side of the rectangular barrier. That this effect cannot be adapted into superluminal signaling is made clear in the second paper, which identifies the early-time tail of the tunneling-time distribution as controlling when the first detection event most likely occurs. It is shown that the MacColl–Hartman superluminality essentially fades away at early times.

The purpose of the present paper is to connect those tunneling calculations to the Sauter–Schwinger effect [5,6,7]. The barriers employed in the previous simulations are supercritical: their heights are large enough that bound states of the Dirac Hamiltonian are embedded in the opposite-energy continuum [8,9]. In that regime the external field does not merely scatter the incident electron; it also destabilizes the vacuum and creates electron–positron pairs [5,6,7]. Therefore, if one wishes to interpret the earlier superluminal tunneling calculations physically, it is essential to compute the pair-production densities that accompany the propagation. This paper has three main goals. First, we derive the electron and positron densities for the case of an initial one-electron state in the presence of a time-dependent external potential. The derivation is based on the Bogoliubov transformation of the field operator and makes transparent the role of positive- and negative-energy projections. We adopt the standard external-field QED mode-expansion/Bogoliubov framework [10,11]. The operator and mode-expansion viewpoint taken here is also close in spirit to Refs. [12,13]. Second, we present numerical 1+1-dimensional densities for a supercritical super-Gaussian barrier, which produces superluminal tunneling of an electron. Third, we compare the numerical pair number with a simple semiclassical resonance model and then show how that model is easily extended to 1+3 dimensions.

Throughout we use relativistic units with ℏ=c=m=1 unless noted otherwise.

## 2. Field-Operator Description and Density Formulas

Consider the Dirac field operator expanded in positive- and negative-energy eigenstates of the free Hamiltonian,

(1) $$\hat{\Psi }\left(r,t\right)=\sum_{q}{\hat{b}}_{q}\left(t\right)\;\psi_{q}^{+}\left(r\right)+\sum_{q}{\hat{d}}_{q}^{†}\left(t\right)\;\psi_{q}^{-}\left(r\right),$$

where ${\hat{b}}_{q}$ annihilates an electron and ${\hat{d}}_{q}^{†}$ creates a positron. Here the compound index q denotes momentum and spin; i.e., $q\equiv (p,m_{s})$. The coefficient functions in the field-operator expansion are the positive- and negative-energy eigenstates of the free Dirac Hamiltonian, here labeled by momentum and spin. These are $L^{2}$-normalized spinor-valued functions of the space variable, *z*. For convenience, momentum is treated as discrete, and the associated eigenstates are normalized to unity. Time evolution in the external field induces a Bogoliubov transformation [10,11],

(2) $$\begin{array}{cc} {\hat{b}}_{q}\left(t\right) & =\sum_{q^{\prime }}\left[\langle \psi_{q}^{+}|\psi_{q^{\prime }}^{+}\left(t\right)\rangle {\hat{b}}_{q^{\prime }}+\langle \psi_{q}^{+}|\psi_{q^{\prime }}^{-}\left(t\right)\rangle {\hat{d}}_{q^{\prime }}^{†}\right], \end{array}$$

(3) $$\begin{array}{cc} {\hat{d}}_{q}^{†}\left(t\right) & =\sum_{q^{\prime }}\left[\langle \psi_{q}^{-}|\psi_{q^{\prime }}^{+}\left(t\right)\rangle {\hat{b}}_{q^{\prime }}+\langle \psi_{q}^{-}|\psi_{q^{\prime }}^{-}\left(t\right)\rangle {\hat{d}}_{q^{\prime }}^{†}\right]. \end{array}$$

These expressions for the time-dependent creation and annihilation operators follow from expanding the field operator in free positive- and negative-energy modes while allowing the corresponding mode functions to evolve in the presence of the external potential. The coefficients are overlap amplitudes between free positive- and negative-energy states and the time-evolved states. In the language of external-field QED, they are Bogoliubov coefficients connecting the in and out mode decompositions [10,11]. Because we use $L^{2}$-normalized free Dirac modes, we can easily define how to project spinor states onto the positive- and negative-energy subspaces. Specifically, the time-evolved negative-energy state projected onto the positive-energy subspace is

(4) $$\psi_{q}^{+-}\left(r,t\right)=\sum_{q^{\prime }}\psi_{q^{\prime }}^{+}\left(r\right)\langle \psi_{q^{\prime }}^{+}|\psi_{q}^{-}\left(t\right)\rangle .$$

Similarly the positive-energy state projected onto the negative-energy subspace is written as

(5) $$\psi_{q}^{-+}\left(r,t\right)=\sum_{q^{\prime }}\psi_{q^{\prime }}^{-}\left(r\right)\langle \psi_{q^{\prime }}^{-}|\psi_{q}^{+}\left(t\right)\rangle .$$

Now the vacuum densities are succinctly expressed in the following form:

(6) $$\begin{array}{cc} \rho_{\mathrm{vac}}^{e}\left(r,t\right) & =\sum_{q}{\left|\psi_{q}^{+-}\left(r,t\right)\right|}^{2}, \end{array}$$

(7) $$\begin{array}{cc} \rho_{\mathrm{vac}}^{p}\left(r,t\right) & =\sum_{q}{\left|\psi_{q}^{-+}\left(r,t\right)\right|}^{2}. \end{array}$$

There is one caveat, however. Because the potential turns on at some time and presumably turns off at some (perhaps much) later time, these densities are not unique local observables of the background-field theory. As such, these densities are quasi-particle electron and positron densities, defined with respect to the free-particle Dirac Hamiltonian energy eigenstates. They only become true densities after the external potential has been switched off, or in spatial regions where the potential is negligible.

Equations (6) and (7) give the Sauter–Schwinger electron and positron densities (subject to the above caveat). They are written here in the standard external-field mode-function (Furry-picture) representation of pair creation [11]. Equation (6) shows that the electron density originates from negative-energy states that acquire a positive-energy component under the action of the external field. Equation (7) is the corresponding charge-conjugate statement for positrons.

Now, let an additional electron be present initially in the state

(8) $$\varphi^{+}\left(r\right)=\sum_{q}c_{q}\;\psi_{q}^{+}\left(r\right).$$

Let $\varphi^{++}\left(r,t\right)$ denote the time-evolved one-electron wavefunction projected onto the positive-energy subspace and $\varphi^{-+}\left(r,t\right)$ the same state projected onto the negative-energy subspace. Then the exact field-theoretic density formulas derived from the Bogoliubov transformation reduce to the following compact expressions:

(9) $$\begin{array}{cc} \rho_{1e}^{e}\left(r,t\right) & =\rho_{\mathrm{vac}}^{e}\left(r,t\right)+{\left|\varphi^{++}\left(r,t\right)\right|}^{2}, \end{array}$$

(10) $$\begin{array}{cc} \rho_{1e}^{p}\left(r,t\right) & =\rho_{\mathrm{vac}}^{p}\left(r,t\right)-{\left|\varphi^{-+}\left(r,t\right)\right|}^{2}. \end{array}$$

These are derived in Appendix A. Equation (9) has a simple interpretation: the positive-energy component of the propagated incident electron adds incoherently to the vacuum-produced electron density. Equation (10) contains the corresponding Pauli-blocking correction for positrons. If the evolving electron acquires a negative-energy component, then that part of the Dirac sea is already occupied and cannot contribute independently to pair creation. A positron-hole effect associated with an initially present electron is also discussed in Ref. [13].

The formulas above neglect explicit interactions between the incident electron and the Sauter–Schwinger electrons and positrons. For the barriers considered here, this approximation is expected to be qualitatively reliable. A mean-field Hartree–Fock estimate implies only small potential energy corrections compared with the barrier height of 7.5 ${\mathrm{mc}}^{2}$ used below. For example, the mean-field interaction energy of two electrons, one reduced Compton wavelength apart (the distance unit used below), with the electrons represented by Gaussian wavepackets with a width less than one reduced Compton wavelength, is about 1/137 ${\mathrm{mc}}^{2}$. Since the electronic wavefunctions appearing below are all much wider than one reduced Compton wavelength, all mean-field energy corrections are even smaller. Also, because of the high velocities considered below, the overlap time during which the wavepacket and newly created particles occupy the same spatial region is very short. For the same reason, direct electron–positron annihilation is expected to be negligible on the time scales resolved in the present simulations.

## 3. Model System and Numerical Setup

In the earlier papers [3,4] we used rectangular potentials because they produce the largest and least ambiguous MacColl–Hartman effect. This is because, in the case of a smooth potential, each energy component of an incident wavepacket experiences a different tunneling space-interval. In this paper, since we are accounting for the Sauter–Schwinger effect, potentials with discontinuities are not acceptable—they are associated with the points of an infinite electric field. Here, we use a super-Gaussian that is smooth but otherwise much like a rectangular potential. Higher order super-Gaussians are even more rectangular-like. However, they require more grid points and time steps to achieve convergence. The external scalar potential used below is the super-Gaussian barrier,

(11) $$V\left(z,t\right)=f\left(t\right)\;V_{0}\exp\left[-{\left(z/w\right)}^{4}\right],$$

with $V_{0}=7.5$ and w=5. In the numerical simulations reported below, the barrier is turned on linearly,

(12) $$f\left(t\right)=\left\{\begin{array}{cc} t/10, & 0\leq t\leq 10,\\ 1, & t>10, \end{array}\right.$$

so that there are initially no Sauter–Schwinger electrons or positrons. This ramp avoids introducing a pre-existing pair density at t=0 and makes the buildup of the densities easier to interpret. The one-electron part of the state is the same type of Gaussian wavepacket used in our previous tunneling studies.

In a static supercritical potential the relevant spectral structure is determined by the Klein zone, where positive-energy electron states overlap negative-energy positron states [8,9]. Supercritical resonances arise when quasibound levels embedded in this overlap region acquire finite widths [8,9]. Those widths govern the temporal growth of pair production. The spatially dependent densities obtained from the full wavepacket evolution shown in Section 4—obtained using the split operator and fast Fourier transform method [14,15,16]—provide a direct way to assess whether the spontaneous pair-production component remains dynamically subdominant or becomes comparable to the incident electron density.

In Section 4, the initial wavepacket (Equation (8)) is a Gaussian localized far to the left of the barrier, as in the earlier papers [3,4]. The wavepacket is initially localized at z=−120, with a width of 8 and an incoming velocity of 0.992. All computations presented below were performed using Python 3.10 with the numpy library.

## 4. Spatial Densities in the Wavepacket Calculations

**Figure 1:**
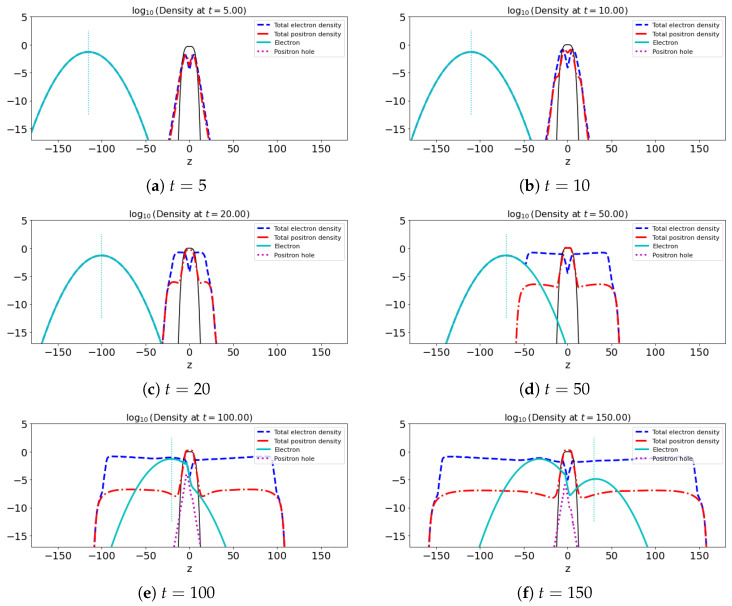
Logarithmic electron and positron densities for the fourth-order super-Gaussian barrier with $V_{0}=7.5$ and w=5. The distance and time units are the reduced Compton wavelength, 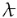 =ℏ/(mc), and 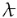/c, respectively. Blue dashed: total electron density; red dashed and dotted: total positron density; cyan solid: one-electron contribution $|\varphi^{++}{|}^{2}$; magenta dotted: positron-hole term $|\varphi^{-+}{|}^{2}$; black: barrier profile. The potential is ramped linearly from t=0 to t=10. The initial Gaussian wavepacket has incoming velocity 0.992. The vertical cyan line in each panel indicates light-speed travel of the center of the initial wavepacket.

Figure 1 shows the electron and positron densities at six representative times, together with the propagated one-electron contribution and the positron hole term appearing in Equations (9) and (10). Several features are immediately apparent.

At early times, while the barrier is still being turned on, both the electron (blue, dashed) and positron (red, dashed and dotted) Sauter–Schwinger densities remain small. By t=10 the central structure has become visible, but the total densities are still localized near the barrier. As time increases to t=20 and t=50, the total electron and positron densities develop shoulders outside the central region, with the positron-density shoulders being much smaller. At later times, t=100 and t=150, the total electron density contains a substantial spatially extended component associated with the propagated incident electron, whereas the positron density remains dominated by the pair-created contribution localized in and near the supercritical region. In the corresponding positron picture, the positrons see a well, while the electrons see a barrier.

Figure 1 also shows the tunneling electron density (cyan, solid) and associated hole term, $|\varphi^{-+}{|}^{2}$, which reduces the positron density (magenta, dotted). The initial velocity of the incoming wavepacket is 0.992. The hole term remains concentrated near the barrier center for all times. The tunneling component of the one-electron density emerges as a separate peak at t=150. The vertical cyan line in that panel indicates light-speed travel from the initial wavepacket position. The tunneling peak lies slightly to the right of this line, indicating effective superluminality. At the same time, the tunneled one-electron component is much smaller than the accompanying Sauter–Schwinger background and is therefore effectively masked by it. Reducing the barrier height would also require reducing the initial wavepacket velocity in order to maintain tunneling rather than over-the-barrier transmission. The reduction in pair-production background needed to isolate the tunneled component appears to require an initial wavepacket velocity too small to display effective superluminality. However, there is a simple solution. In Section 5, a semiclassical model is developed that shows that Sauter–Schwinger pair production saturates at longer times. Consequently, if we delay the introduction of the incoming relativistic electron until most of the Sauter–Schwinger electron density has drifted far to the right or left—away from the barrier neighborhood—then the density associated with the tunneling component of that electron can exceed the Sauter–Schwinger electron density and is not masked as above. This is elaborated in Section 5.

## 5. Semiclassical Resonance Picture of the Sauter–Schwinger Effect

A useful interpretation of the pair-production dynamics is obtained from the electron and positron pictures of the supercritical barrier [8,9]. The associated resonance-width description of vacuum decay also admits a semiclassical WKB interpretation [17]. This viewpoint is closely related to the worldline-instanton approach to Sauter–Schwinger pair production, in which the relevant contribution to the effective action is obtained by a saddle-point, or steepest-descent, approximation to a first-quantized path-integral representation [18,19,20]. The usefulness of such semiclassical descriptions in the present context can be understood from the fact that the dominant phases and tunneling exponents are controlled by dimensionless actions, in units of *ℏ*, accumulated with relativistic velocity over length and time scales of several reduced Compton units.

**Figure 2:**
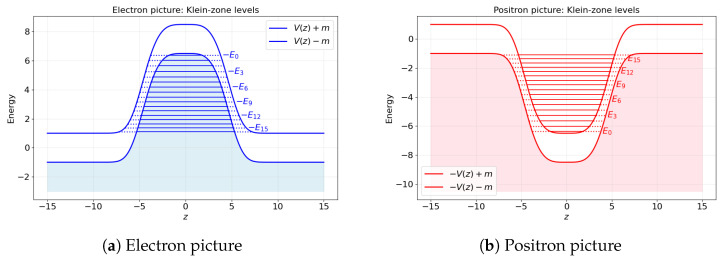
Klein-zone levels for the super-Gaussian barrier in the electron and positron pictures. The resonance structure is most transparent in the region where the positive- and negative-energy continua overlap.

**Figure 3:**
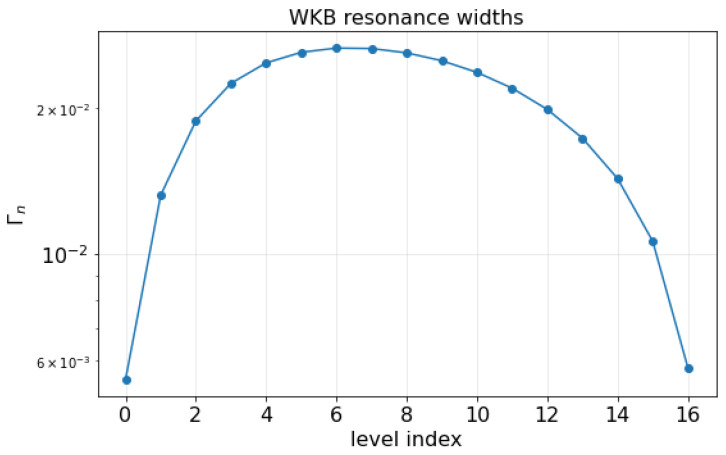
Supercritical resonance parameters used in the semiclassical model. The widths peak for the middle modes because the associated imaginary WKB actions are smallest there.

Here, we view electron–positron pair production as resulting from resonance energy levels in the Klein zone. In the electron picture, the Klein zone is the energy range shared by V(z)−m and V(z)+m. In the positron picture, the Klein zone is the range shared by −V(z)−m and −V(z)+m. These two representations are shown in Figure 2. The resonance energies are the WKB levels in the well picture. The associated WKB widths, resulting from the gap between the two wells, are plotted in Figure 3.

The widths are largest for the middle modes, as levels near the bottom and top of the Klein zone have larger imaginary actions because the classically forbidden regions become wider there. By contrast, intermediate levels have the shortest tunneling paths in the effective one-dimensional problem and therefore the largest escape widths. Since the widths set the rate at which a given resonance contributes to pair creation, the middle levels dominate the temporal buildup of the pair number [8,9].

For a set of resonance energies $E_{n}$ and widths $\Gamma_{n}$, a simple cycle-resolved WKB model for the one-dimensional pair number is given by

(13) $$N^{1D}\left(t\right)=\sum_{n}\left[1-\exp(-\Gamma_{n}t)\right].$$

**Figure 4:**
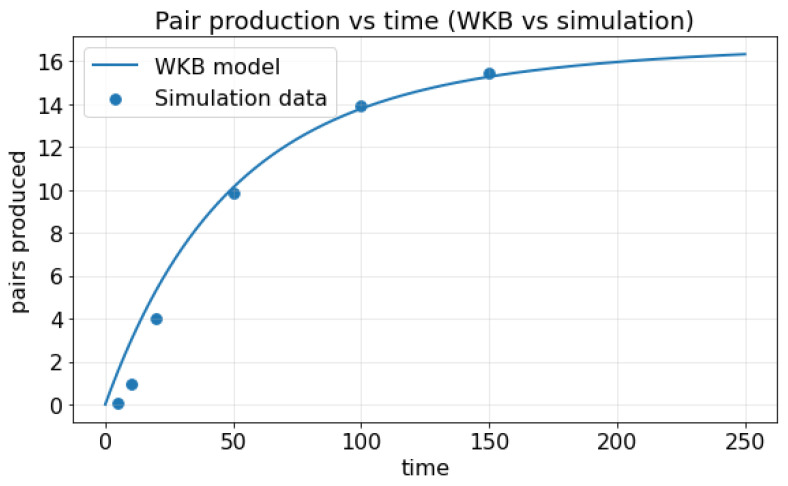
Total pair number versus time: simulation data compared with the semiclassical WKB model. The WKB curve does not include the linear ramp from t=0 to 10, which accounts for the largest discrepancy at early times.

The widths are modeled as $\Gamma_{n}=\left(2/T_{n}\right)\exp\left(-2S_{n}\right)$ where $T_{n}$ is the classical period in the well and $S_{n}$ is the imaginary action associated with passage across the forbidden region between the positron Dirac sea and the well resonance state. This is the usual semiclassical structure of a tunneling rate: an attempt frequency multiplied by a barrier-penetration probability, adapted here to the supercritical resonance problem [8,17]. Our width model is just the product of the probability of tunneling per encounter with the well edge and the rate of such encounters. Initially there is no barrier, and hence no occupied supercritical resonances. Subsequently, the resonances fill according to a first-order kinetics ansatz introduced here, motivated by the resonance widths and by Pauli saturation of each quasibound channel [8,9]. The resulting pair number is compared with the simulation data in Figure 4. For the simulation pair number data, the Sauter–Schwinger electron and positron densities were integrated separately. These numbers always agree, as the formalism strictly enforces particle production in pairs. The simulation data and the model are a good match over the full growth region, becoming better after the end of the turn-on ramp. The main systematic difference occurs at early times because the numerical calculation uses the linear ramp while the WKB model shown here is based on the static barrier without that initial turn-on.

Now that we have some confidence in the semiclassical model, the model can be further developed to give the time evolution of the Sauter–Schwinger electron density. We only consider electron density here, because it is the electron density that masks the superluminal tunneling component of the incoming electron. Most of the Sauter–Schwinger positrons stay in the barrier (a well for them). The positrons that escape the barrier have much lower density than the associated electrons and will leave the neighborhood of the barrier just like the electrons. The positrons remaining in the barrier have the effect of lowering the effective barrier height. We can choose the initial barrier height to be larger in order to compensate for this effect and get a desired effective barrier height.

We develop a heuristic model for the Sauter–Schwinger electron density, as a more rigorous model is beyond the scope of this paper. The model is supported by agreement with the simulation results.

The model posits that Sauter–Schwinger electrons appear at the turning points of the barrier resonances with a rate of $\Gamma_{n}e^{-\Gamma_{n}t}/2$–half of the total rate applies to each turning point, $z_{n}$ and $-z_{n}$. These emerging electrons are represented by width 1, $L^{1}$-normalized Gaussians, $g(z-z_{n})$ and $g(z+z_{n})$. These narrow Gaussians replace delta functions, implied by the underlying premise of the model, to facilitate numerical computation. In the model, the total electron density at position *z* and time *t* is expressed as

(14) $$\rho_{e}\left(z,t\right)=\frac{1}{2}\sum_{n}\int_{0}^{t}dt^{\prime }\;\Gamma_{n}e^{-\Gamma_{n}t^{\prime }}\left\{g\;\left[z-z_{n}-v_{n}\left(t-t^{\prime }\right)\right]+g\;\left[z+z_{n}+v_{n}\left(t-t^{\prime }\right)\right]\right\}.$$

**Figure 5:**
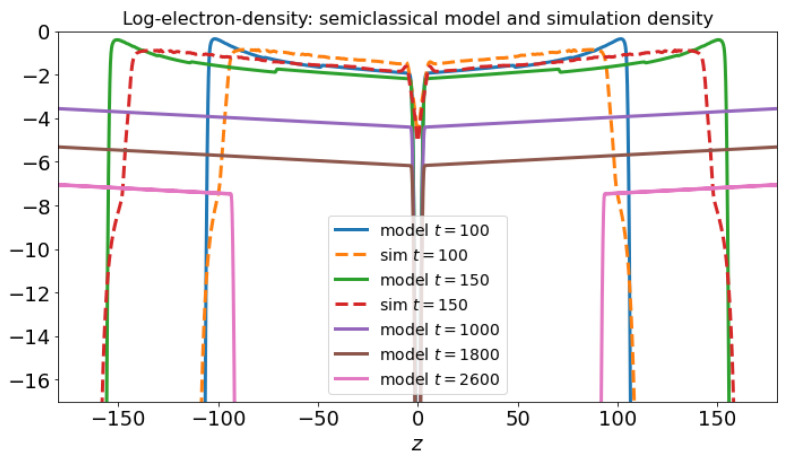
Semiclassical model of the Sauter–Schwinger electron density for t=100, 150, 1000, 1800 and 2600. For the first two times, simulation results are included for comparison (dashed lines).

Figure 5 shows semiclassical densities compared to simulation results from Section 4 for t=100 and 150. Since these are log-plots, the model cannot be considered to be a good approximation to the simulation results. However, it does capture the general shape and size of the Sauter–Schwinger electron density. This is good enough for our purposes. Also shown are model results for t=1000, 1800 and 2600. Generating simulation data for these long times would be challenging. They would require implementing absorbing boundaries, and using a much larger grid, in addition to the many more time steps. In contrast, the semiclassical model easily provides an estimate of the size of the Sauter–Schwinger density at these long times, and shows how the density decreases with time once all Klein zone resonances are filled by positrons. Specifically, the roughly flat broad distribution decreases exponentially (note the even spacing of the three long-time data sets) until the two sides of the distribution separate, and move away to the left and right–see in the t=2600 data set. The latter effect arises because there is a lowest velocity of the emitted electrons determined by the lowest barrier resonance energy. These observations support our conclusion that an incoming relativistic electron, introduced well after the onset of the barrier, could produce a tunneling component not masked by Sauter–Schwinger electron density.

## 6. Extension from 1+1 to 1+3 Dimensions

The one-dimensional semiclassical model extends naturally to three spatial dimensions by integrating over the conserved transverse momentum. For a static scalar potential depending only on *z*, the transverse momentum enters only through the effective mass

(15) $$m_{⊥}=\sqrt{m^{2}+p_{⊥}^{2}}.$$

**Figure 6:**
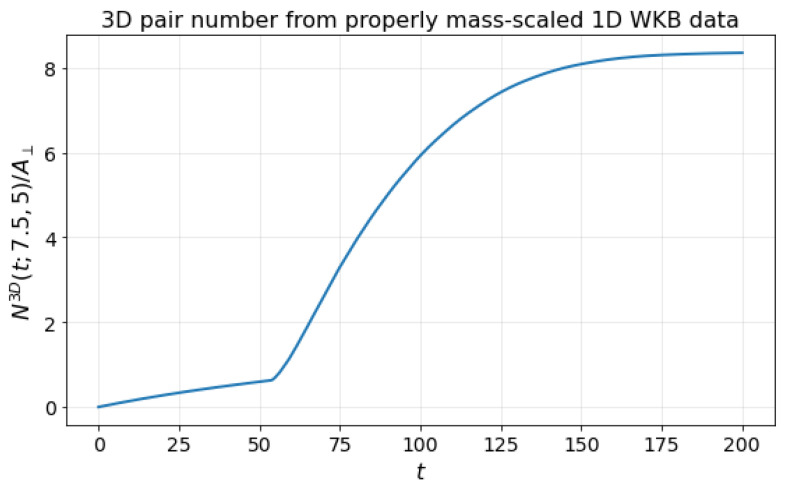
Three-dimensional pair density per unit transverse area obtained from integrating interpolated one-dimensional WKB data.

Therefore the three-dimensional pair density per unit transverse area, $A_{⊥}$, is

(16) $$\begin{array}{cc} \frac{N^{3D}\left(t;V_{0},w,m\right)}{A_{⊥}} & =2\int \frac{d^{2}p_{⊥}}{{\left(2\pi \right)}^{2}}\;N^{1D}\left(t;V_{0},w,m_{⊥}\right)\\ \; & =\frac{1}{2\pi }\int_{m}^{\infty }m_{⊥}\;N^{1D}\left(t;V_{0},w,m_{⊥}\right)\;dm_{⊥}\\ \; & =\frac{1}{2\pi }\int_{m}^{\infty }m_{⊥}\;N^{1D}\left(m_{⊥}t;V_{0}/m_{⊥},m_{⊥}w,1\right)\;dm_{⊥}. \end{array}$$

where the factor of 2 accounts for spin. In practice, the integral is truncated at the largest $m_{⊥}$ for which the barrier remains supercritical. In the units used here, the transverse-area unit is $A_{⊥}=$ 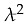, and the quantity plotted in Figure 6 is the pair number per 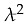.

The integral in Equation (16) is evaluated by exploiting the scaling of the one-dimensional model with respect to the mass parameter, as seen in the last line of the equation. Figure 6 shows the three-dimensional density obtained by integrating interpolated mass-scaled one-dimensional data. In particular, we compute $N^{1D}\left(m_{⊥}t;7.5/m_{⊥},m_{⊥}5,1\right)$ for a grid of $m_{⊥}$ values, then interpolate these values to get a common grid of times for all $m_{⊥}$. For $m_{⊥}t$ beyond the largest time considered, we use the long time limiting value of $N^{1D}$. As expected, the three-dimensional density grows monotonically and saturates at long times, but the saturation value now represents a surface density rather than a pure number.

## 7. Discussion

The results presented here sharpen the interpretation of the earlier superluminal tunneling calculations. The apparent time advance of the transmitted wavepacket is a property of the positive-energy projection of the propagated one-electron state, but the same external field also produces a non-negligible Sauter–Schwinger background. In a supercritical barrier the two phenomena are inseparable: the wavepacket dynamics and the vacuum instability are governed by the same Klein-zone resonance structure.

The explicit density formulas, Equations (9) and (10), show that the relationship between the incident electron and the pair-created particles is simple at the level of one-body densities. The electron density contains an additive propagated-electron term on top of the vacuum contribution, whereas the positron density is reduced by Pauli blocking associated with the negative-energy component of the incident electron. This provides a transparent physical interpretation of the numerical density plots.

The semiclassical analysis explains why the pair number grows on the observed time scale and why the middle resonance levels dominate. The remaining early-time mismatch between the semiclassical curve and the simulation is not a failure of the resonance picture; it is mainly the consequence of the linear turn-on used in the full wavepacket computation. Incorporating the turn-on profile into the semiclassical model would be a natural refinement.

From a broader perspective, the present work reinforces the main message of our earlier papers [3,4]. Even though the tunneling component of a Dirac wavepacket may exhibit effective superluminal propagation, this does not enable superluminal signaling. The first-click distribution remains causal, and the inclusion of the Sauter–Schwinger background would not alter that conclusion. What it does alter is the physical bookkeeping: once the barrier is recognized as supercritical, any complete description must include the accompanying electron and positron densities.

Claims of effective superluminal transit associated with tunneling, and MacColl–Hartman effective speedup, have been discussed from several different viewpoints [2,21,22]. A common interpretation emphasizes pulse reshaping by the barrier. Specifically, the transmitted packet is said to be formed preferentially from the leading tail of the incident packet, so that the apparent speedup is not a genuine traversal effect [22,23]. While such interpretations capture important features of wave-packet filtering, they do not seem to us to fully account for the phenomenon studied here. In particular, if a smooth incident packet is decomposed into smooth front, middle, and rear subpackets, each transmitted component itself exhibits the MacColl–Hartman saturation and their own effective speedup. In this sense, the transmitted packet is not simply selected from the leading edge of the incident packet; rather, the tunneling component arises from the entire incident wavepacket. Some other conservative interpretations connect the tunneling group delay to stored energy, dwell time, or self-interference, and similarly avoid assigning a literal superluminal transit velocity to the particle [22,24]. Another approach distinguishes the group velocity, which may be superluminal in tunneling or anomalously dispersive systems, from the front velocity, which is usually identified with the propagation velocity of a nonanalytic signal front and is taken to remain luminal [23,25,26]. This distinction is important, but its direct application to tunneling wave packets is not without difficulty. A physically relevant wavepacket must be smooth and reasonably narrow in momentum space. Introducing a sharp front or discontinuity produces large high-momentum components, that cross above the barrier rather than tunnel through it. Furthermore, for positive-energy relativistic wavepackets, strict compact support is itself problematic. Hegerfeldt’s theorem, discussed in the Dirac context by Thaller, implies that a strictly localized positive-energy state immediately develops nonzero tails arbitrarily far away [27,28].

Our interpretation is therefore closer in spirit to that advocated by Recami and co-workers, who regarded superluminal tunneling group delays as physically meaningful manifestations of superluminal propagation, while still distinguishing them from straightforward superluminal signalling [29,30]. Recami and collaborators went further, developing an “extended relativity” framework in which a tachyonic sector of always-superluminal particles is admitted within a generalized Lorentz-invariant theory [29,31]. That broader program is beyond the scope of the present work. The present paper instead concerns ordinary fermionic particles governed by the Dirac equation. The point is not to introduce tachyonic particles, but to show that ordinary relativistic tunneling can display MacColl–Hartman-type effective superluminal transit without implying superluminal signalling.

## 8. Conclusions

We have shown that the supercritical barriers used in earlier relativistic tunneling calculations necessarily generate Sauter–Schwinger electron–positron pairs. Using the Bogoliubov transformation for the Dirac field, we derived compact formulas for the electron and positron densities in the presence of one additional electron. These formulas were then illustrated numerically for a linearly turned-on super-Gaussian barrier of height $V_{0}=7.5$.

The numerical densities and the semiclassical resonance model tell a consistent story. Pair production is controlled by a finite set of Klein-zone resonances, with the middle modes contributing most strongly because their WKB widths are largest. The one-dimensional pair number obtained from the full wavepacket calculation is reproduced well by the semiclassical model aside from the expected early-time difference caused by the turn-on ramp. Finally, the mass-scaling construction provides a practical route from one to three dimensions, where the relevant observable is the pair density per unit transverse area.

These results place the earlier superluminal tunneling calculations in a more complete physical context. Superluminal tunneling and vacuum pair creation are not competing descriptions of the same barrier; they are simultaneous consequences of the same supercritical Dirac dynamics.

## Data Availability

The data presented in this study are available on reasonable request from the corresponding author.

## Appendix A. Derivation of the One-Electron Density Formulas

For the one-electron initial state of Equation (8), the electron and positron densities may be written as

(A1) $$\begin{array}{cc} \rho_{1e}^{e}\left(r,t\right) & =\langle \;\langle \mathrm{vac}|\sum_{q}c_{q}^{*}\;{\hat{b}}_{q}{\hat{\Psi }}^{+†}\left(r,t\right){\hat{\Psi }}^{+}\left(r,t\right)\sum_{q^{\prime }}c_{q^{\prime }}\;{\hat{b}}_{q^{\prime }}^{†}|\mathrm{vac}\rangle \;\rangle , \end{array}$$

(A2) $$\begin{array}{cc} \rho_{1e}^{p}\left(r,t\right) & =\langle \;\langle \mathrm{vac}|\sum_{q}c_{q}^{*}\;{\hat{b}}_{q}{\hat{\Psi }}^{-}\left(r,t\right){\hat{\Psi }}^{-†}\left(r,t\right)\sum_{q^{\prime }}c_{q^{\prime }}{\hat{b}}_{q^{\prime }}^{†}|\mathrm{vac}\rangle \;\rangle , \end{array}$$

where ${\hat{\Psi }}^{+}$ and ${\hat{\Psi }}^{-}$ denote the positive- and negative-energy parts of the field operator, i.e., the first and second terms on the right-hand side of Equation (1), respectively. Inserting Equations (2) and (3) then yields the desired expressions. Equation (2) leads to the following form for the electron density:

(A3) $$\begin{array}{cc} \rho_{1e}^{e}\left(r,t\right)=\sum_{q,q_{1},q_{2},q_{3},q_{4},q^{\prime }} & c_{q}^{*}c_{q^{\prime }}\langle \;\langle \mathrm{vac}|{\hat{b}}_{q}\psi_{q_{1}}^{+†}\left(r\right)\left[\langle \psi_{q_{2}}^{+}\left(t\right)|\psi_{q_{1}}^{+}\rangle {\hat{b}}_{q_{2}}^{†}+\langle \psi_{q_{2}}^{-}\left(t\right)|\psi_{q_{1}}^{+}\rangle {\hat{d}}_{q_{2}}\right]\\ \; & \left[\langle \psi_{q_{3}}^{+}|\psi_{q_{4}}^{+}\left(t\right)\rangle {\hat{b}}_{q_{4}}+\langle \psi_{q_{3}}^{+}|\psi_{q_{4}}^{-}\left(t\right)\rangle {\hat{d}}_{q_{4}}^{†}\right]\psi_{q_{3}}^{+}\left(r\right){\hat{b}}_{q^{\prime }}^{†}|\mathrm{vac}\rangle \;\rangle . \end{array}$$

Expanding out the bracketed factors gives two terms that make a nonzero contribution to the density. They are the terms that include the factors (inside the vacuum expectation), ${\hat{b}}_{q}{\hat{d}}_{q_{2}}{\hat{d}}_{q_{4}}^{†}{\hat{b}}_{q^{\prime }}^{†}$ and ${\hat{b}}_{q}{\hat{b}}_{q_{2}}^{†}{\hat{b}}_{q_{4}}{\hat{b}}_{q^{\prime }}^{†}$. Specifically,

(A4) $$\begin{array}{cc} \rho_{1e}^{e}\left(r,t\right)=\sum_{q,q_{1},q_{2},q_{3},q_{4},q^{\prime }} & c_{q}^{*}c_{q^{\prime }}\psi_{q_{1}}^{+†}\left(r\right)\psi_{q_{3}}^{+}\left(r\right)[\langle \psi_{q_{2}}^{-}\left(t\right)|\psi_{q_{1}}^{+}\rangle \langle \psi_{q_{3}}^{+}|\psi_{q_{4}}^{-}\left(t\right)\rangle \\ \; & \times \langle \;\langle \mathrm{vac}|{\hat{b}}_{q}{\hat{d}}_{q_{2}}{\hat{d}}_{q_{4}}^{†}{\hat{b}}_{q^{\prime }}^{†}|\mathrm{vac}\rangle \;\rangle +\langle \psi_{q_{2}}^{+}\left(t\right)|\psi_{q_{1}}^{+}\rangle \langle \psi_{q_{3}}^{+}|\psi_{q_{4}}^{+}\left(t\right)\rangle \\ \; & \times \langle \;\langle \mathrm{vac}|{\hat{b}}_{q}{\hat{b}}_{q_{2}}^{†}{\hat{b}}_{q_{4}}{\hat{b}}_{q^{\prime }}^{†}|\mathrm{vac}\rangle \;\rangle ], \end{array}$$

or

(A5) $$\begin{array}{cc} \rho_{1e}^{e}\left(r,t\right) & =\sum_{q,q_{1},q_{2},q_{3}}{\left|c_{q}\right|}^{2}\psi_{q_{1}}^{+†}\left(r\right)\psi_{q_{3}}^{+}\left(r\right)\langle \psi_{q_{2}}^{-}\left(t\right)|\psi_{q_{1}}^{+}\rangle \langle \psi_{q_{3}}^{+}|\psi_{q_{2}}^{-}\left(t\right)\rangle \\ \; & +\sum_{q,q_{1},q_{3},q^{\prime }}c_{q}^{*}c_{q^{\prime }}\psi_{q_{1}}^{+†}\left(r\right)\psi_{q_{3}}^{+}\left(r\right)\langle \psi_{q}^{+}\left(t\right)|\psi_{q_{1}}^{+}\rangle \langle \psi_{q_{3}}^{+}|\psi_{q^{\prime }}^{+}\left(t\right)\rangle . \end{array}$$

Since $\sum_{q}{\left|c_{q}\right|}^{2}=1$, the first term takes the form

(A6) $$\sum_{q_{2}}{\left|\sum_{q_{3}}\psi_{q_{3}}^{+}\left(r\right)\langle \psi_{q_{3}}^{+}|\psi_{q_{2}}^{-}\left(t\right)\rangle \right|}^{2}=\rho_{\mathrm{vac}}^{e}\left(r,t\right).$$

Using Equation (4), this term is seen to be the vacuum electron density of Equation (6). The second term takes the form

(A7) $${\left|\sum_{q_{3}}\psi_{q_{3}}^{+}\left(r\right)\langle \psi_{q_{3}}^{+}|\sum_{q^{\prime }}c_{q^{\prime }}\psi_{q^{\prime }}^{+}\left(t\right)\rangle \right|}^{2}={\left|\varphi^{++}\left(r,t\right)\right|}^{2},$$

which is just the modulus squared of the propagated one-electron wavefunction projected onto the positive-energy subspace. Altogether, the total electron density reduces to Equation (9):

$$\rho_{1e}^{e}\left(r,t\right)=\rho_{\mathrm{vac}}^{e}\left(r,t\right)+{\left|\varphi^{++}\left(r,t\right)\right|}^{2}.$$

Insertion of Equation (3) into the negative-energy field operator leads to the expression for the positron density. As above, we get the product of two bracketed factors. Upon expanding this product, only one term makes a nonzero contribution to the vacuum expectation value. Specifically, we get

(A8) $$\rho_{1e}^{p}\left(r,t\right)=\sum_{q,q_{1},q_{2},q_{3},q_{4},q^{\prime }}c_{q}^{*}c_{q^{\prime }}\psi_{q_{1}}^{-†}\left(r\right)\psi_{q_{3}}^{-}\left(r\right)\langle \psi_{q_{2}}^{+}\left(t\right)|\psi_{q_{1}}^{-}\rangle \langle \psi_{q_{3}}^{-}|\psi_{q_{4}}^{+}\left(t\right)\rangle \langle \;\langle \mathrm{vac}|{\hat{b}}_{q}{\hat{b}}_{q_{2}}{\hat{b}}_{q_{4}}^{†}{\hat{b}}_{q^{\prime }}^{†}|\mathrm{vac}\rangle \;\rangle .$$

As above, the vacuum expectation collapses the multiple sum. There are, however, two resulting contractions: either $q_{2}=q_{4}$ and $q=q^{\prime }$, or $q=q_{4}$ and $q_{2}=q^{\prime }$. The latter contraction carries an additional minus sign, arising from the interchange of anticommuting electron creation and annihilation operators. Specifically, ${\hat{b}}_{q}{\hat{b}}_{q_{2}}{\hat{b}}_{q_{4}}^{†}{\hat{b}}_{q^{\prime }}^{†}=-{\hat{b}}_{q}{\hat{b}}_{q_{4}}^{†}{\hat{b}}_{q_{2}}{\hat{b}}_{q^{\prime }}^{†}$. This minus sign is the origin of the Pauli-blocking correction in the positron density. The result is

(A9) $$\begin{array}{cc} \rho_{1e}^{p}\left(r,t\right)=\sum_{q,q_{1},q_{2},q_{3}} & {\left|c_{q}\right|}^{2}\psi_{q_{1}}^{-†}\left(r\right)\psi_{q_{3}}^{-}\left(r\right)\langle \psi_{q_{2}}^{+}\left(t\right)|\psi_{q_{1}}^{-}\rangle \langle \psi_{q_{3}}^{-}|\psi_{q_{2}}^{+}\left(t\right)\rangle \\ \; & -\sum_{q,q_{1},q_{2},q_{3}}c_{q}^{*}c_{q_{2}}\psi_{q_{1}}^{-†}\left(r\right)\psi_{q_{3}}^{-}\left(r\right)\langle \psi_{q_{2}}^{+}\left(t\right)|\psi_{q_{1}}^{-}\rangle \langle \psi_{q_{3}}^{-}|\psi_{q}^{+}\left(t\right)\rangle . \end{array}$$

Using Equations (5) and (7), the above positron density simplifies to Equation (10):

$$\rho_{1e}^{p}\left(r,t\right)=\rho_{\mathrm{vac}}^{p}\left(r,t\right)-{\left|\varphi^{-+}\left(r,t\right)\right|}^{2}.$$
